# Effects of Combined Dietary Chromium(III) Propionate Complex and Thiamine Supplementation on Insulin Sensitivity, Blood Biochemical Indices, and Mineral Levels in High-Fructose-Fed Rats

**DOI:** 10.1007/s12011-012-9515-5

**Published:** 2012-10-16

**Authors:** Ewelina Król, Zbigniew Krejpcio, Sławomir Michalak, Rafał W. Wójciak, Paweł Bogdański

**Affiliations:** 1 Department of Human Nutrition and Hygiene, Poznan University of Life Sciences, 31 Wojska Polskiego, 60-624 Poznan, Poland; 2 Department of Neurochemistry and Neuropathology, Poznan University of Medical Sciences, 49 Przybyszewskiego, 60-355 Poznan, Poland; 3 Department of Clinical Psychology, Poznan University of Medical Sciences, 70 Bukowska, 60-812 Poznan, Poland; 4 Department of Internal Medicine, Metabolic Disorders and Hypertension, Poznan University of Medical Sciences, 84 Szamarzewskiego, 60-569 Poznan, Poland

**Keywords:** Chromium(III) propionate complex, Thiamine, Supplementation, Insulin resistance, Rats

## Abstract

Insulin resistance is the first step in glucose intolerance and the development of type 2 diabetes mellitus, thus effective prevention strategies should also include dietary interventions to enhance insulin sensitivity. Nutrients, such as microelement chromium(III) and thiamine, play regulatory roles in carbohydrate metabolism. The objective of this study was to evaluate the insulin-sensitizing potential of the combined supplementary chromium(III) propionate complex (CrProp) and thiamine in insulin resistance animal model (rats fed a high-fructose diet). The experiment was carried out on 40 nine-week-old male Wistar rats divided into five groups (eight animals each). Animals were fed ad libitum: the control diet (AIN-93 M) and high-fructose diets with and without a combination of two levels of CrProp (0.1 and 1 mg Cr/kg body mass/day) and two levels of thiamine (0.5 and 10 mg/kg body mass/day) for 8 weeks. At the end of the experiment rats were sacrificed to collect blood and internal organs for analyses of blood biochemical and hematologic indices as well as tissular microelement levels that were measured using appropriate methods. It was found that both supplementary CrProp and thiamine (given alone) have significant insulin-sensitizing and moderate blood-lipid-lowering properties, while the combined supplementation with these agents does not give synergistic effects in insulin-resistant rats. CrProp given separately increased kidney Cu and Cr levels, while thiamine alone increased hepatic Cu contents and decreased renal Zn and Cu contents.

## Introduction

Major causes of the observed epidemic of obesity include overnutrition, sedentary lifestyles, and a lack of physical activity that bring about serious health problems such as metabolic disorders, insulin resistance, type 2 diabetes, and cardiovascular disease [1]. It is predicted that by the year 2030 over 366 million people worldwide will be afflicted by type 2 diabetes [2]. The insulin resistance, defined as an impaired responsiveness of the body to insulin, is a prediabetic stage associated with obesity, leading to type 2 diabetes [3]. Studies have shown that insulin resistance is observed long before the development of diabetes, thus it is believed that early identification and treatment of individuals with prediabetes can delay the progression of full-blown diabetes and related complications [4]. Diet and exercise can to some extent attenuate insulin resistance, but they are often inefficient in bringing about significant improvements. For this reason pharmacological medications (e.g., thiazolinodiones and biguanides) are usually applied. However, these drugs can cause serious side effects. Therefore increasing efforts have been made to search for bioactive compounds from dietary sources that are able to improve insulin action and blood glucose and lipid levels.

Among dietary components involved in glucose metabolism, microelement chromium(III) is considered as a potential agent able to attenuate insulin resistance and its consequences.

Chromium(III) has a documented role in carbohydrate, lipid, and protein metabolism [5]; however, the mechanisms of its action on the molecular level are not fully understood. A recent broad overview of the biological actions of Cr(III) was excellently presented by Hua et al. [6], therefore, it will not be repeated in this article. In short, many clinical studies [7–9] showed a significant improvement of glucose tolerance after Cr(III) supplementation in type 2 diabetes. However, there are also other trials that did not confirm a positive effect of Cr(III) supplementation in diabetes [10–12].

Irrespective of controversial scientific opinions, various Cr(III) compounds (mostly Cr(III) tris picolinate, CrPic) have been advertised, marketed, and used as very popular dietary supplements to decrease body mass gain, improve glycemic control, or reduce appetite. However, the results of a recent model study performed on rats raised some doubts on the essentiality of Cr for mammals [13].

If Cr(III) is not an essential element for mammals, but at certain dosages improves impaired glucose and lipid homeostasis, its action could be called “pharmacological” at best [14].

Thiamine is another nutritional factor of interest in the context of glucose metabolism and insulin action. This vitamin is a coenzyme for transketolase, pyruvate dehydrogenase, and the alpha-ketoglutrate dehydrogenase complex [15]; it affects blood glucose level by the hexosamine pathway [16], prevents hyperglycemia by reduction of cell replication and proliferation, and decreases the production of advanced glycation end products [17]. Another mechanism, by which administration of thiamine (T) or its derivates can influence carbohydrates metabolism, is improving the pentose phosphate pathway [18]. Mutation of the thiamine transporter SLC19A2 gene located on chromosome 1q32 is associated with diabetes mellitus, sensorineural deafness, and megaloblastosis [19, 20]. A low plasma thiamine concentration was observed in types 1 and 2 diabetic patients, and this parameter was also proposed to be a marker of microvascular diabetic complications [21]. Evidence from experimental and clinical studies suggests that metabolism and clearance of thiamine is disturbed in diabetes, leading to tissue-specific thiamine deficiency in the kidney and other sites of development of vascular complications.

Kohda et al. [22] investigated the mechanism of diabetes-induced pyruvate dehydrogenase (PDH) inhibition, and the effect of thiamine in both in vivo and in vitro experiments. Treatment of rats with thiamine significantly, although partially, recovered streptozotocin (STZ)-induced reductions in mitochondrial PDH activity.

Recently, Pacal et al. [23] revealed the role of thiamine status and genetic variability in transketolase and other pentose phosphate cycle enzymes in the progression of diabetic nephropathy in a group of 240 diabetic subjects. The results supported the role of ‘functional’ thiamine deficiency in the development of hyperglycemia-related pathology.

Thiamine supplementation prevented the development of early-stage nephropathy in diabetic rats and reversed increased urinary albumin excretion in patients with type 2 diabetes and microalbuminuria in two recent clinical trials [24]. In animal models of type 1 (streptozotocin-injected) and type 2 diabetes (leptin-receptor mutant mice) benfotiamine prevented cardiomyopathy [25].

As it was shortly presented above, the mechanisms of Cr(III) and thiamine action in glucose metabolism are surely different. In our previous study [26], we provided evidence that supplementary chromium(III) propionate complex (CrProp), given in dosages of 1 and 5 mg/kg body mass/day for 8 weeks, is able to ameliorate insulin resistance symptoms in high-fructose fed Wistar rats.

In this study, using the insulin resistance animal model (rats fed high-fructose diet), we tested the hypothesis whether the combined administration of these agents could increase the efficacy of insulin action and whether a synergistic effect may occur.

## Materials and Methods

### Animals and Diets

Nine-week-old 40 male Wistar rats were purchased at the Licensed Laboratory Animals Breeding Center (Poznan, Poland). Animals were housed at constant temperature (21 ± 2 °C), humidity (55–60 %) and with a 12 h/12 h day/night cycle. After 5 days of adaptation period animals were divided into five groups of eight rats each, and kept individually in semi-metabolic cages. Diets used in the experiment were composed, according to the AIN-93 M recommendations [27] (Table 1). The high-fructose diets were obtained from the basal AIN-93 diet, by replacement of wheat starch with fructose (up to 60 %). Animals were fed ad libitum: the control diet (AIN-93 M) and high-fructose diets (HF) with variable levels of Cr(III) (as the chromium(III) propionate complex and thiamine: HF (Cr and T adequate, 1 and 5 mg/kg diet, respectively; equals to 0.1 mg Cr and 0.5 mg T/kg body mass per day, respectively), HF+Cr (supplemented with 10 mg Cr/kg diet, equals to 1 mg Cr/kg body mass per day), HF+T (supplemented with T 100 mg/kg diet, equals to 10 mg/kg body mass per day) and HF+T+Cr (supplemented with Cr and T, 10 and 100 mg/kg diet; equals to 1 and 10 mg/kg body mass per day) for 8 weeks.

**Table 1:** Composition of diets used in experiment

Ingredients	Diet				
	Control (C)	High fructose (HF)	High fructose + Cr (HF+Cr)	High fructose + thiamine (HF+T)	High fructose + thiamine + Cr (HF+T+Cr)
Casein (g/kg)	200	200	200	200	200
Soybean oil (g/kg)	70	70	70	70	100
Wheat starch (g/kg)	535	35	35	35	35
Fructose (g/kg)	0	600	600	600	600
Sucrose (g/kg)	100	0	0	0	0
Potato starch (g/kg)	50	50	50	50	50
Vitamin mix (g/kg)	10	10	10	10	10
Mineral mix (g/kg)	35	35	35	35	35
Cr^a^ (mg/kg)	1	1	10	1	10
Thiamine^b^ (mg/kg)	5	5	5	100	100

^a^CrProp was added to mineral mix

^b^Thiamine hydrochloride was added to vitamin mix

Food intake was monitored daily and body mass gain weekly.

### Test Chemicals

Thiamine hydrochloride was purchased from Sigma-Aldrich (USA), while Cr(III) propionate cation (CrProp) in the form of nitrate salt (chemical formula [Cr_3_O(O_2_CCH_2_CH_3_)_6_(H_2_O)_3_]^+^(NO_3_) was synthesized at the laboratory of the Department of Product Ecology, the Poznan University of Economics, according to the method described previously by Earnshaw et al. [28]. The content of elemental Cr was 21.5 % and it has been determined by the AAS method (spectrometer AAS-3 with BC correction, Zeiss, Germany).

### Data Collection

At the end of the experiment, after 16 h fasting, rats were anesthetized with an intraperitoneal thiopental injection (40 mg/kg body mass) and blood for biochemical tests was collected from the aorta to test tubes containing heparin as a coagulant. Additionally, inner organs (liver, kidneys, heart, spleen, pancreas, and testes) were removed for appropriate biochemical tests. Organs were washed in saline, weighed, and stored at −20 °C until analyzed. All the procedures used in this study were approved by the Animal Bioethics Committee of Poznan, Poland (Approval # 37/2007).

### Laboratory Analyses

#### Blood Biochemistry

The laboratory methods used to determine blood serum indices: glucose, total LDL and HDL cholesterols; triacylglycerols; activity of ALT and AST enzymes; and total protein, urea, and creatinine levels were described previously [26]. Plasma insulin concentration was measured by the RIA method using a kit specific for rats (Linco Research, St. Charles, MO, USA), while tumor necrosis factor alpha (TNF-α) and interleukin-2 (IL-2) levels by ELISA kits (R&D Systems, USA). Total antioxidant status (TAS) was assessed spectrophotometrically using a commercial kit (Randox, UK).

Erythrocyte transketolase (ETK) activity and the effect of adding thiamine pyrophosphate (% TPP effect) were measured by method described previously [29].

The efficacy of insulin resistance was characterized by the homeostasis model assessment (HOMA) indices [30].

$$ \mathrm{HOMA}-\mathrm{IR}={{{\left( {\mathrm{Fasting}\,\mathrm{Glucose}\left[ {{{\mathrm{mmol}} \left/ {{\mathrm{d}{{\mathrm{m}}^3}}} \right.}} \right]\times \mathrm{Fasting}\,\mathrm{Insulin}\left[ {{{\mathrm{mIU}} \left/ {{\mathrm{d}{{\mathrm{m}}^3}}} \right.}} \right]} \right)}} \left/ {22.5 } \right.} $$

### Microelement Determinations

Prior to analysis the rats’ tissues were digested in 65 % (*w*/*w*) spectra pure HNO_3_ (Merck) in the Microwave Digestion System (MARS 5, CEM). Thereafter, the concentrations of Fe, Zn, and Cu in the mineral solutions were measured by the flame-AAS method (spectrometer AAS-3, Zeiss, with BC, Germany), while the content of Cr was determined by the graphite furnace AAS method (spectrometer AA EA 5, with BC, Jenoptic, Germany).

The accuracy of quantitative determinations of Fe, Zn, Cu, and Cr was confirmed by a simultaneous analysis of the certified reference material (Pig Kidney BCR® No. 186, Brussels, fortified with the Cr standard).

### Statistical Analysis

All results are presented as means ± standard deviation. Significance of differences in means were calculated using the one-way ANOVA and Tukey’s test. Means were considered statistically different at *p* < 0.05. Main and interaction effects of experimental factors (Cr(III) and thiamine) were determined by two-way analysis of variance. All calculations were made using the STATISTICA (ver. 7.0) program (StatSoft, Inc. Tulsa).

## Results

The present study is first to our knowledge that evaluates the combined effects of supplementary CrProp and thiamine on insulin resistance and related blood parameters, as well as tissular microelement levels in the insulin-resistant rat model (induced by a high-fructose diet).

In a previous article [26] the effects of supplementary CrProp on carbohydrate and lipid metabolism in the insulin resistance rat model was presented. Some of those results are referenced in this study.

**Fig. 1:**
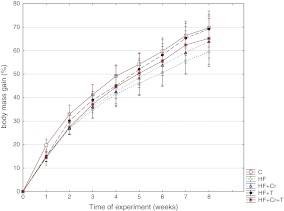
Body mass gain expressed in percentage of initial body mass during experimental period

The effects of a high-fructose diet as well as supplementary Cr(III)Prop (0.1 and 1 mg/kg body mass/day) and thiamine (0.5 and 10 mg/kg body mass/day) on overall nutritional indices of rats fed high-fructose diet were presented on Fig. 1 and Table 2.

**Table 2:** Effect of Cr(III), thiamine, and thiamine combined with Cr(III) overall growth indices in rats

Index	Experimental group				
	C	HF	HF+T	HF+Cr	HF+T+Cr
Food intake (g/day)	18.49 ± 0.63	19.95 ± 0.63	20.82 ± 0.88	20.60 ± 1.39	20.45 ± 1.49
Relative body weight gain (%)	69.21 ± 5.12	59.25 ± 5.89	63.06 ± 7.63	67.94 ± 8.81	62.42 ± 8.14
Body mass/body length ratio (g/cm)	14.26 ± 0.53	14.65 ± 0.78	15.62 ± 0.85	15.44 ± 0.25	15.24 ± 0.95

*C* group fed control diet, *HF* group fed high-fructose diet, *HF*+*Cr* group fed high-fructose diet supplemented with Cr(III), *HF*+*T* group fed high-fructose diet supplemented with thiamine, *HF*+*Cr*+*T* group fed high-fructose diet supplemented with Cr(III) and thiamine

As it can be seen on Fig. 1, the high-fructose diet tended to decrease relative body mass gain in rats in comparison to the control group; however, due to the variability of individual responses no significant differences were observed. Neither supplementary CrProp nor thiamine (alone and combined) influenced food intake or overall nutritional outcomes in rats (Table 2).

High-fructose feeding, as expected, induced insulin resistance and related blood indices, such as increased serum insulin level, the HOMA-IR index, and serum triacylglycerol concentration by 188, 240, and 63 %, respectively. However, it did not influence parameters of oxidative stress (TAS) or inflammation biomarkers (TNF-α and IL-2) (Table 3). Both supplementary CrProp and thiamine significantly attenuated insulin resistance biomarkers in rats fed the HF diet. Additionally, erythrocyte transketolase activity, that is often used as a biomarker of thiamine status, was significantly increased in the HF group (by 41 %), only slightly in the HF+T group (by 31 %), while surprisingly enough, it was decreased in the HF+Cr group (by 32 %).

**Table 3:** Effect of Cr(III), thiamine, and thiamine combined with Cr(III) supplementation on biochemical indices of rats (mean ± SD)

Parameter	Experimental group				
	C	HF	HF+T	HF+Cr	HF+T+Cr
Glucose concentration (mmol/dm^3^)	6.34 ± 0.58^ab^	7.38 ± 0.75^b^	7.17 ± 0.57^b^	6.95 ± 0.89^ab^	6.17 ± 0.61^a^
Insulin concentration (mIU/dm^3^)	18.74 ± 7.25^a^	53.23 ± 14.52^b^	29.59 ± 14.47^ab^	32.03 ± 9.51^ab^	27.1 ± 11.1^a^
HOMA-IR index	5.28 ± 1.96^a^	17.46 ± 5.03^b^	9.51 ± 3.12^ab^	10.13 ± 3.64^a^	7.28 ± 3.30^a^
Total cholesterol concentration (mmol/dm^3^)	2.12 ± 0.21	2.47 ± 0.30	2.30 ± 0.31	2.25 ± 0.21	2.31 ± 0.36
HDL cholesterol concentration (mmol/dm^3^)	1.44 ± 0.15	1.63 ± 0.19	1.48 ± 0.18	1.58 ± 0.21	1.62 ± 0.22
LDL cholesterol concentration (mmol/dm^3^)	0.49 ± 0.08	0.58 ± 0.13	0.44 ± 0.09	0.52 ± 0.13	0.47 ± 0.08
Triacylglycerols concentration (mmol/dm^3^)	0.35 ± 0.14^ab^	0.58 ± 0.13^b^	0.53 ± 0.21^ab^	0.50 ± 0.15^ab^	0.33 ± 0.05^a^
TAS (mmol/dm^3^)	0.93 ± 0.12	1.03 ± 0.16	0.86 ± 0.03	1.01 ± 0.09	0.92 ± 0.09
TNF-α (ng/dm^3^)	167 ± 18	163 ± 27	165 ± 14	159 ± 25	159 ± 12
IL-2 (ng/dm^3^)	374 ± 22	397 ± 48	354 ± 44	352 ± 32	363 ± 45
Erythocytes transketolase activity (U/g Hb)	0.81 ± 0.33^ab^	1.14 ± 0.24^b^	1.06 ± 0.26^b^	0.55 ± 0.24^a^	0.98 ± 0.26^ab^

Each value is expressed as mean ± standard deviation for eight rats per experimental group. Values in the row without common superscripts differ significantly at *p* < 0.05

*C* group fed control diet, *HF* group fed high-fructose diet, *HF*+*Cr* group fed high-fructose diet supplemented with Cr(III), *HF*+*T* group fed high-fructose diet supplemented with thiamine, *HF*+*Cr*+*T* group fed high-fructose diet supplemented with Cr(III) and thiamine

The results of two-way analysis of variance are presented in Table 6. As it can be seen, only supplementary CrProp (1 mg Cr/kg body mass/day) had a small, but significant blood glucose lowering effect (by 9 %). Both supplementary CrProp and thiamine (independently) decreased blood insulin concentration (by 27 and 28 %, respectively), the HOMA-IR index (by 36 and 40 %), and triacylglycerol concentration (by 36 and 19 %).

As it could be expected, supplementary thiamine (10 mg/kg body mass/day) significantly increased blood ETK activity (by 40 %). Surprisingly enough, supplementary CrProp decreased ETK activity (by 30 %).

Since insulin resistance is a metabolic state linked with an increased oxidative stress and inflammation, serum total antioxidative status and serum interleukin IL-2 concentration were measured. It was found that serum TAS was significantly decreased (by 13 %) only by supplementary thiamine. No interaction effects of experimental factors were observed for these indices.

These results indicate that both supplementary CrProp and thiamine can improve insulin action and glucose utilization independently, but no synergistic effects occur during co-administration of these agents.

In the case of a prolonged drug or supplementary treatment in diabetes or any other diseases, it is necessary to consider possible adverse effects. Thiamine is a water-soluble vitamin, practically non-toxic. There are no reports available on adverse effects from consumption of excess thiamine by ingestion of food and supplements. So far no tolerable upper intake level (UL) was established for thiamine.

Although Cr(III) compounds are rather of low acute toxicity, in some cases excessive dosages can do harm in various organs and systems [31]. For this reason it is necessary to monitor biomarkers of general toxicity. As can be seen in Table 4, neither HF diet nor supplementary CrProp and thiamine affected toxicity parameters in rats, except serum ALT level, that was significantly increased (by 27 %), while both supplements attenuated these changes. An interaction effect for serum ALT level was observed. When Cr was given at adequate levels with supplementary thiamine it decreased serum ALT activity.

**Table 4:** Effect of Cr(III), thiamine, and thiamine combined with Cr(III) supplementation on general toxicity indices of rats (mean ± SD)

Blood index	Experimental group				
	C	HF	HF+T	HF+Cr	HF+T+Cr
ALT (U/dm^3^)	22.73 ± 3.25^a^	28.71 ± 3.20^b^	23.71 ± 3.09^a^	21.88 ± 2.75^a^	25.00 ± 3.16^ab^
AST (U/dm^3^)	108.71 ± 13.15	114.07 ± 16.15	105.43 ± 7.23	106.3 ± 10.4	111.29 ± 11.40
Total protein (10^−2^ kg/dm^3^)	5.87 ± 0.14	5.98 ± 0.22	6.03 ± 0.25	6.03 ± 0.18	6.01 ± 0.20
Creatinine (μmol/dm^3^)	36.39 ± 3.54	37.09 ± 3.51	36.24 ± 3.54	33.64 ± 3.54	35.36 ± 5.31
Urea (mmol/dm^3^)	5.32 ± 0.64	5.01 ± 0.90	5.42 ± 0.76	5.39 ± 1.04	5.26 ± 0.46

Each value is expressed as mean ± standard deviation for eight rats per experimental group. Values in the row without common superscripts differ significantly at *p* < 0.05

*C* group fed control diet, *HF* group fed high-fructose diet, *HF*+*Cr* group fed high-fructose diet supplemented with Cr(III), *HF*+*T* group fed high-fructose diet supplemented with thiamine, *HF*+*Cr*+*T* group fed high-fructose diet supplemented with Cr(III) and thiamine

Supplementary minerals and vitamins given in excessive dosages can affect mineral status. The effects of high-fructose diet and supplementary CrProp and thiamine on tissular Fe, Zn, Cu, and Cr levels are presented in Table 5. The high-fructose diet slightly increased liver Fe content and significantly decreased liver Cu level (by 13 %). Supplementary CrProp and thiamine (given separately) decreased liver Fe (both by 25 %), while the combined administration of these compounds normalized liver Fe in rats. On the other hand, supplementary CrProp and thiamine (both separately and combined) normalized liver Cr level in rats.

**Table 5:** The contents of Fe, Zn, Cu, and Cr in liver and kidney of rats

Index	Experimental group				
	C	HF	HF+T	HF+Cr	HF+T+Cr
Liver (μg/g d.m.)					
Fe	317 ± 41^b^	353 ± 46^b^	263 ± 23^a^	264 ± 50^a^	307 ± 53^ab^
Zn	100.1 ± 8.1	103.4 ± 9.6	94.8 ± 5.4	101.3 ± 8.1	96.5 ± 14.3
Cu	22.8 ± 2.4^b^	19.7 ± 2.5^a^	23.8 ± 2.3^b^	21.7 ± 1.8^ab^	21.1 ± 2.5^ab^
Cr	0.52 ± 0.09	0.48 ± 0.08	0.51 ± 0.12	0.61 ± 0.16	0.62 ± 0.09
Kidney (μg/g d.m.)					
Fe	345.9 ± 33	322.0 ± 32	339.3 ± 36	305.0 ± 18	329.3 ± 53
Zn	82.4 ± 7.1	83.0 ± 7.5	74.7 ± 4.6	82.24 ± 6.86	76.2 ± 6.3
Cu	39.6 ± 6.3^b^	35.5 ± 6.4^ab^	32.5 ± 6.6^a^	38.84 ± 8.12^b^	33.1 ± 2.5^a^
Cr	0.56 ± 0.15^a^	0.56 ± 0.17^a^	0.57 ± 0.12^a^	0.84 ± 0.08^b^	0.83 ± 0.16^b^

Each value is expressed as mean ± standard deviation for eight rats per experimental group. Values in the row without common superscripts differ significantly at *p* < 0.05

*C* group fed control diet, *HF* group fed high-fructose diet, *HF*+*Cr* group fed high-fructose diet supplemented with Cr(III), *HF*+*T* group fed high-fructose diet supplemented with thiamine, *HF*+*Cr*+*T* group fed high-fructose diet supplemented with Cr(III) and thiamine

The high-fructose diet did not induce significant changes in kidney microelement concentrations.

Supplementary CrProp, as expected, brought about an increase of kidney Cr level (by 47 %), but it did not affect tissular Fe and Zn status in rats. Supplementary thiamine markedly decreased kidney Cu content.

Analysis of variance showed that supplementary CrProp significantly elevated kidney Cu and Cr levels (by 10 and 47 %, respectively), while supplementary thiamine markedly increased liver Cu content (by 11 %), whereas it decreased kidney Zn and Cu concentrations (by 7 and 13 %, respectively; Table 6).

**Table 6:** Main and interaction effects of supplementation with thiamine and CrProp in rats fed high-fructose diets

Index	Main effects		Interaction effects
	Cr level (mg/kg b.w./day) 0.1 vs 1.0	Thiamine level (mg/kg b.w/.day) 0.5 vs 10	Cr × thiamine p
Glucose concentration	7.21 ± 0.7	7.13 ± 0.7	NS
(mmol/dm^3^)	6.56 ± 0.7*	6.71 ± 0.8	
Insulin concentration	41.3 ± 23.6	39.7 ± 19.8	NS
(mIU/dm^3^)	30.2 ± 10.3*	28.3 ± 12.1*	
HOMA-IR index	13.7 ± 4.39	14.0 ± 4.45	NS
	8.71 ± 3.97*	8.39 ± 3.21*	
Triacylglycerols concentration	0.55 ± 0.16	0.53 ± 0.11	NS
(mmol/dm^3^)	0.43 ± 0.11*	0.43 ± 0.18*	
Erythocytes transketolase activity	1.09 ± 0.37	0.77 ± 0.38	NS
(U/g Hb)	0.76 ± 0.36*	1.08 ± 0.39*	
ALT	26.2 ± 3.9	25.1 ± 4.5	**
(U/dm^3^)	23.3 ± 3.3*	24.4 ± 3.1	
TAS	0.95 ± 0.14	1.02 ± 0.12	NS
(mmol/dm^3^)	0.97 ± 0.10	0.89 ± 0.07*	
Liver Fe	305 ± 59.7	305 ± 67.2	***
(μg/g d.m.)	286 ± 54.3	285 ± 45.4	
Liver Cu	21.8 ± 3.27	20.3 ± 2.8	*
(μg/g d.m.)	21.0 ± 2.56	22.5 ± 2.7*	
Kidney Zn	77.8 ± 6.9	81.5 ± 7.1	NS
(μg/g d.m.)	79.2 ± 7.1	75.5 ± 5.4*	
Kidney Cu	33.4 ± 5.7	37.6 ± 5.9	NS
(μg/g d.m.)	36.8 ± 5.3*	32.8 ± 4.5*	
Kidney Cr	0.57 ± 0.14	0.70 ± 0.13	NS
(μg/g d.m.)	0.84 ± 0.12*	0.70 ± 0.14	

Only parameters for which significant effects were detected are presented

*p < 0.05; **p < 0.01; ***p < 0.001 (statistically significant differences)

Besides, interaction effects were observed for liver Fe and Cu levels. When Cr was given at adequate levels with supplementary thiamine it decreased liver Fe level, while increased liver Cu content.

## Discussion

Insulin resistance preceded by inflammation increases the risk of diabetes, hypertension, and cardiovascular diseases. A decreased insulin sensitivity manifests itself in a reduced insulin-stimulated glucose uptake, reduced insulin-suppression of endogenous glucose production, reduced antilipolysis, decreased insulin-induced vasodilation, dyslipidemia, and platelet hyperaggregability [32]. Additionally, decreased insulin sensitivity, due to the interruption in the biological rhythm of the key metabolic pathways, affects food intake control, digestion, transport, and absorption of nutrients [33].

Various Cr(III) compounds as dietary supplements and therapeutic agents have been studied extensively for their insulin sensitizing, glucose and lipid lowering, and anti-diabetic potential in animal models and diabetic patients in the last decades. The most studied compound for this purpose was Cr tris picolinate (CrPic), although giving inconsistent results. Another promising form of Cr is CrProp, introduced by Vincent and co-workers [34–39].

Clodfelder et al. [37] reported that Cr_3_O(O_2_CCH_2_CH_3_)_6_(H_2_O)_3_)^+^ is absorbed with a very high efficiency of 40–60 %, while popular Cr supplements such as CrCl_3_, Cr(III) nicotinate, or CrPic are absorbed at only 0.5–1.3 % of the gavaged dose. The difference in the degree of absorption is readily explained by the stability and solubility of the cation in the physiological milieu. The detailed mechanisms of anti-diabetic action of CrProp have not been fully elucidated.

The therapeutic properties of CrProp in relation to its anti-diabetic, insulin-sensitizing potential were discussed in other publications [12, 26, 38] and will not be repeated in this article.

The importance of thiamine for the endocrine and exocrine function of pancreas β-cells was described in the 1970s and 1980s. Thiamine deficiency was shown to impair the insulin synthesis and secretion [40, 41]. On the other hand, a reduction of thiamine transport across the intestine has been reported in insulin deficiency states [18]. It has been suggested that thiamine deficiency may occur in diabetes [21]. However, to the authors knowledge there is no information whether insulin resistance and hyperinsulinemia can influence thiamine status.

In this study, rats fed the high-fructose diet developed insulin resistance and hyperinsulinemia, which did not affect erythrocytes transketolase activity. However, ETK is not a sensitive biomarker of thiamine status, thus acceleration of its activity by thiamine pyrophosphate (TPP) may give more accurate results. The enhancement of ETK activity (after adding TPP) indicates thiamine deficiency. Also other biomarkers of thiamine status (e.g., erythrocyte thiamine and its phosphorylated esters, urinary thiamine excretion [42]) should be measured to give more insight into the relationship between thiamine and insulin resistance.

However, the purpose of this study was not to search for this relationship, but rather to evaluate the therapeutic potential of supplementary thiamine (alone) and combined with CrProp.

In the literature there is some information concerning the therapeutic potential of thiamine and its analogue in hyperglycemic states. For example, Berrone et al. [43] showed that human umbilical vein endothelial cells and bovine retinal pericytes treated in vitro with thiamine and benfotiamine (a synthetic analogue of thiamine) reduced activation of the polyol pathway of glucose metabolism and increased transketolase expression in the presence of hyperglycemia.

In an animal (rat) model of insulin-deficient diabetes, Karachalias et al. [44] found that long-term (24 weeks) thiamine and benfotiamine supplementation decreased HbA1C and prevented an increased urinary excretion of protein glycation, oxidation, and nitration adducts, thus these agents reversed early-stage diabetic nephropathy. Gonzalez-Ortiz et al. in a pilot study [45] reported that drug-naïve type 2 diabetic patients administered 150 mg of thiamine per day for 4 weeks had slightly decreased blood glucose and leptin concentrations. However, this intervention did not influence HbA1C, blood lipids, or inflammation markers (IL-6, TNF-α, and adiponectin).

In diabetic STZ-injected rats, high-dose thiamine (70 mg/kg of diet/day) given for 24 weeks decreased blood total cholesterol and triacylglycerol levels [16].

Both thiamine and Cr(III) compounds are considered as potential antioxidants. In the rat liver microsomes thiamine decreased lipid peroxidation due to a direct interaction with free radicals and hydroperoxides [46]. Also better absorbed lipid-soluble analogs of thiamine, i.e., benfotiamine, reduced oxidative stress in rat and porcine kidney cells as well as in rats and humans [47–50].

In this study, supplementary thiamine (10 mg/kg body mass/day) showed a moderate antioxidant capacity, as it reduced serum TAS level by 13 % in rats fed a high-fructose diet.

In insulin resistance oxidative stress and increased inflammation usually occur, which induces the expression of specific cytokines. Tumor necrosis factor alpha, was the first proinflammatory cytokine found to induce insulin resistance [51, 52]. Later such ability was confirmed also for other cytokines, such as IL-6, resistin, monocyte chemoattractant protein-1, angiotensinogen, visfatin, retinol-binding protein-4 [53–56]. According to a hypothesis presented by Penntinen [57], an increased plasma IL-2 concentration brings about a reduction of bioavailability of insulin-like growth factor-1, by reducing the production of androgenic hormones, thus contributing to insulin resistance.

In this study, neither thiamine alone nor combined with CrProp affected serum TNF-α or IL-2 levels. Similarly, in a study by Gonzalez-Ortiz et al. [45] thiamine administration did not improve IL-6, TNF-α, or adiponectin levels in drug-naïve diabetics.

Anti-inflammatory properties of Cr(III) compounds were examined by Jain and co-workers [58, 59]. The authors found that Cr(III) (administered in niacinate or dinicocysteinate) improved inflammatory status as determined by interleukin IL-6, TNF-α, C-reactive protein, monocyte chemotactic protein-1, and intercellular adhesion molecule 1 (ICAM-1) in diabetic rats.

Supplementary minerals, including Cr, can affect mineral status, due to possible interactions with other macro- and microelements at absorption, transport, metabolism, and other levels. For example, Cr(III) and Fe can compete for binding sites in transferrin. For this reason the question of Cr–Fe interactions should be addressed whenever Cr(III) is to be administrated orally. In our previous study [12], it was shown that supplemental CrProp, given at dosages of 5 mg Cr/kg body mass/day for 8 weeks, decreased the kidney Fe concentration in rats. Also Clodfelder et al. [38] reported that supplementary CrProp (1 mg Cr kg/body mass/day, for 24 weeks) also decreased kidney Fe content in a rat model of early stage of type 2 diabetes. Dogukan et al. [60] observed that high-fat fed streptozotocin-injected rats supplemented with Cr(III) histidinate (8.2 % elemental Cr) in dosages of 110 μg CrHis kg body mass/day for 10 weeks had decreased liver and kidney Cu content with a parallel increase in Zn levels in both organs. Similar results were previously noticed in another study of this team with regard to young and pregnant rabbits [61].

The mechanism of thiamine–mineral interactions is unknown and the literature data is scant. Although in this study neither thiamine nor thiamine–Cr(III)Prop supplementation affected tissular zinc contents, in some studies such a relationship was mentioned. For example, Agte et al. [62] investigated the effect of water-soluble vitamins on in vitro zinc uptake in human erythrocytes in Zn deficiency, normal and excess conditions. It was found that in Zn deficiency an addition of thiamine to media enhanced Zn uptake, while under normal level and excess of zinc there was no interaction. On the other hand, thiamine enhanced zinc transport in a model of the small intestine (Caco-2 cells) only in a condition without oxidative stress [63]. However, these studies were performed under in vitro conditions, thus due to a lack of endocrine function they cannot mimic in vivo conditions.

Thiamine is a compound containing in its molecule four nitrogen atoms and one sulfur atom, thus it has some metal-chelating potential. It was used in the treatment of metal (Pb and Cd) poisoning [64–66]. For this reason we evaluated the effects of thiamine on tissular (liver and kidney) Fe, Zn, Cu, and Cr levels in rats. It was shown that supplementary thiamine (10 mg/kg body mass/day) increased liver Cu content (by 11 %), while it decreased kidney Zn and Cu levels (7–13 %) in high-fructose fed rats. However, these changes appear to be rather small, thus their significance for the overall mineral status is unclear.

In conclusion, the results of this study confirmed that both supplementary CrProp (1 mg/kg body mass/day) and thiamine (10 mg/kg body mass/day) have comparable insulin-sensitizing and blood-lipid-lowering potential in rats.

Generally, the efficacy of CrProp action seems stronger than that of thiamine, since the dose of the latter applied in the experiment was tenfold higher, to produce similar effects in rats. However, no synergistic effects have been detected. Therefore there is no rationale to advocate the combined CrProp and thiamine supplementation for the prevention and treatment of insulin resistance.
